# Motivational interviewing interactions and the primary health care challenges presented by smokers with low motivation to stop smoking: a conversation analysis

**DOI:** 10.1186/1471-2458-14-1225

**Published:** 2014-11-26

**Authors:** Núria Codern-Bové, Enriqueta Pujol-Ribera, Margarida Pla, Javier González-Bonilla, Silvia Granollers, José L Ballvé, Gemma Fanlo, Carmen Cabezas, ISTAPS Study Group

**Affiliations:** Escola Universitària d’Infermeria i Teràpia Ocupacional de Terrassa, Universitat Autònoma de Barcelona, C/De la Riba, 90, 08221 Terrassa, Spain; Institut Universitari d’Investigació en Atenció Primària Jordi Gol (IDIAP Jordi Gol), Àmbit d’Atenció Primària de Barcelona, Institut Català de la Salut, C/Gran Via Corts Catalanes, 587, àtic, 08007 Barcelona, Spain; Cátedra de recerca qualitativa, Fundació Dr Robert, Universitat Autònoma de Barcelona, C/St. Antoni Maria Claret, 171, 08041 Barcelona, Spain; Equip d’Atenció Primària Sant Just Desvern. SAP Baix Llobregat Centre, Av. Indústria, Sant Just Desvern, 08960 Barcelona, Spain; Equip d’Atenció Primària L’Hospitalet de Llobregat - 7 Florida Nord, C/Parc dels Ocellets, s/n, 08905 Hospitalet de Llobregat, Spain; Equip d’Atenció Primària L’Hospitalet de Llobregat - 11 Gornal, C/Carmen Amaya, 37-39, 08902 Hospitalet de Llobregat, Spain; Departament de Salut. Subdirecció de Prevenció i Promoció de Salut, C/de Roc Boronat, 81-95, 08005 Barcelona, Spain

**Keywords:** Conversation analysis, Communication, Motivational interviewing, Primary health care, Qualitative research, Smoking cessation, Social interaction

## Abstract

**Background:**

Research indicates that one third of smokers have low motivation to stop smoking. The purpose of the study was to use Conversational Analysis to enhance understanding of the process in Motivational Interviewing sessions carried out by primary care doctors and nurses to motivate their patients to quit smoking. The present study is a substudy of the Systematic Intervention on Smoking Habits in Primary Health Care Project (Spanish acronym: ISTAPS).

**Methods:**

Motivational interviewing sessions with a subset of nine participants (two interview sessions were conducted with two of the nine) in the ISTAPS study who were current smokers and scored fewer than 5 points on the Richmond test that measures motivation to quit smoking were videotaped and transcribed. A total of 11 interviews conducted by five primary health care professionals in Barcelona, Spain, were analysed. Qualitative Content Analysis was used to develop an analytical guide for coding transcriptions. Conversation Analysis allowed detailed study of the exchange of words during the interaction.

**Results:**

Motivational Interviewing sessions had three phases: assessment, reflection on readiness to change, and summary. The interaction was constructed during an office visit, where interactional dilemmas arise and can be resolved in various ways. Some actions by professionals (use of reiterations, declarations, open-ended questions) helped to construct a framework of shared relationship; others inhibited this relationship (focusing on risks of smoking, clinging to the protocol, and prematurely emphasizing change). Some professionals tended to resolve interactional dilemmas (e.g., resistance) through a confrontational or directive style. Interactions that did not follow Motivational Interviewing principles predominated in seven of the interviews analysed.

**Conclusions:**

Conversational analysis showed that the complexity of the intervention increases when a health professional encounters individuals with low motivation for change, and interactional dilemmas may occur that make it difficult to follow Motivational Interview principles. Incorporating different forms of expression during the Motivational Interviewing could help to build patient-centred health care relationships and, for patients with low motivation to stop smoking, offer an opportunity to reflect on tobacco use during the office visit. The study findings could be included in professional training to improve the quality of motivational interviewing.

## Background

Tobacco use is a preventable health problem linked to 25% of deaths among adults younger than 65 years in developed countries [1, 2], making it the principal cause of premature death in these populations. In Spain, the percentage of the general population that smokes daily is declining steadily, from 32.1% in 1993 to 24% in 2012 [3]; nonetheless, health problems related to smoking are one of the most common reasons for visits to the health care system in general, and to primary health care (PHC) centres in particular [4]. The PHC setting is the most common resource for smoking cessation attempts [5]. Given that 70% of smokers annually visit a primary care professional, these centres have a strategic role in smoking cessation [6, 7].

A study in Great Britain reported that one third of smokers reported low motivation to stop smoking [8]. Interventions by health professionals improve the likelihood of success. Various meta-analyses have shown that brief advice increases quit attempts by a further 1% to 3% [9, 10].

Another approach used in PHC to motivate individuals who are hesitant to make changes or ambivalent about smoking cessation is Motivational Interviewing (MI), based on the work of Miller and Rollnick [11]. This method has been defined as a collaborative, person-centered style for addressing the problem of ambivalence about change. It is designed to strengthen personal motivation and commitment to a specific goal by eliciting and exploring the individual’s own reasons for change, within a climate of acceptance, empathy, and mutual cooperation, ultimately respecting the individual’s decisions [11]. MI has attracted considerable interest because of evidence that it produces better results than brief advice [12], which constitutes usual care in our PHC context [13]. Meta-analyses of smoking cessation interventions have reported that, compared to brief advice, MI achieves a modest but significant increase in the number of cessation attempts and in abstinence rates. However, the authors recommend caution in interpreting the results because of study limitations: variations in the quality of the study design, inadequate evidence of fidelity to MI principles (which had repercussions for motivation to change), and the possibility of publication biases [14–17].

Numerous studies have evaluated the efficacy of MI, focusing on how to measure MI counsellor fidelity in real-world settings and MI trainings [18–20]. These authors applied behavioural coding of MI sessions with fidelity assessment systems like the Motivational Interviewing Skills Code (MISC) [21] and the Motivational Interviewing Treatment Integrity (MITI) [22, 23]. These instruments identify relational and behavioural characteristics of the therapy sessions for both the counsellor and the patient. Although this line of research is important, another approach is based on conversational analysis (CA) that identifies sequences that can offer deep insights into the interaction between the health professional and the patient. This method focuses on a turn-by-turn analysis, which allows a sequential examination of interactions and could shed greater light on the interpretations and assumptions established by the communication [24], compared to the more established MI coding schemes such as the MITI and MISC.

In the sociological discipline, CA has been used to study the health care interaction as a moment-by-moment production space for “human social life” [25]. This approach emerged from Garfinkel’s ethnomethodology [26] and the ethnomethodological CA approach described by Sacks [27], both of which acknowledge talk-in-interaction as a social reality that occurs as turn-taking.

In recent years, several studies and reviews have been published that uses CA to examine patient-health professional interactions to deliver bad news [28] and offer advice on lifestyle changes [29], for example, with findings that may prove to be key to successful professional practice.

Articles by Maynard & Heritage and Pilnick, Hindarsh & Gill showed the collaborative nature of health care interactions. When individuals are paying attention to the conversation and to the behaviour of the other, one will initiate a sequence and this will become a point of reference for the other, generating the second part of the sequence. In addition, co-constructing the interaction involves the notions held by both participants about the subjects they are discussing, as well as the social context in which the interaction takes place [24, 30].

Another topic of interest in CA is the study of how the office visit is organized, the tasks that are completed, and the dilemmas that arise in the interaction. Mikesell [31] reviewed interaction studies that utilized CA, and reported the major findings that could help to build relationships of support and trust, and substantially improve patient health. The findings suggest that a dynamic, collaborative interaction is key to a positive office intervention. Among the studies reviewed was that of Barry et al. [32], which identified four types of health care interaction, defined by the shared or one-directional use of the Voice of the Lifeworld and the Voice of Medicine (using Misheler’s terms) [33]: a) only the Voice of Medicine is used; b) the Voice of the patient (Lifeworld) is blocked by the Voice of Medicine; c) the Voice of the Lifeworld is ignored, and d) the point of departure is the Voice of the Lifeworld. When patients and health professionals work collaboratively, the outcomes improve; this can be measured by the presence or absence of misunderstandings, adherence to therapy, and each participant’s satisfaction with the interaction [25].

Other significant contributions of the CA approach are found in studies by Pilnick & Coleman of office visits that include smoking cessation interventions. The advice to stop smoking is more effective when the health professional incorporates specific strategies to adjust the conversation to a patient’s needs (negotiation of needs and personalization of the message). The patient is then more likely to adopt an attitude of consent that advances the conversation towards the target [34].

Finally, Coleman et al. analysed interactions that occurred while quit-smoking advice was being given, using an adaptation of CA as their method of analysis. They observed that health professionals had a confrontational reaction when faced with rejection of their advice. They also suggest that smoking cessation counselling aimed at patients with low motivation would have better outcomes if health professionals had more advanced conversational skills [35].

All of these aspects, analysed using CA (collaborative nature of the interaction, use of open-ended questions, negotiation), must be identified within MI sessions because they form part of that interaction style. Therefore, CA allows the analysis of specific practices that may, in our case, make motivating the patient more difficult and provide recommendations about the type of specific actions a health professional should carry out to introduce motivational elements into conversation and to improve patient satisfaction [24, 36].

Only a few studies of smoking cessation interventions have examined brief advice about smoking from the CA perspective; no studies were identified that used CA to examine the MI sessions with patients having low motivation to quit smoking.

For these reasons, the present study aimed to use CA to analyse the structure of MI sessions carried out by primary care doctors and nurses in conversations with patients having low motivation to quit smoking. In addition, we examined the actions of the health professionals during the MI session and assessed the consequences in the patient response. These objectives arose from questions such as, “how is the MI session organized? What do people do to understand each other during the MI? What patterns of interaction are in line with the basic MI principles?

The CA results concerning the encounter between a patient with low motivation to stop smoking, and a health professional conducting the MI session can provide useful knowledge to support the studies of the effectiveness of such interviews [37, 38]. The results can also be used to improve the training offered to health professionals about patient communication, in an effort to advance our knowledge in this field.

## Methods

The present work is a substudy of the Systematic Intervention on Smoking Habits in Primary Health Care Project (The ISTAPS study, Spanish acronym), a multicentre, cluster-randomized clinical trial in Spain [39, 40]. This substudy applied a CA approach [26, 41, 42] to analyse the health care interaction, assessing how the conversation between the health professional and patient was structured during the MI session. This research focused on individuals with low motivation to stop smoking. All participants, both patients and health professionals, were concurrently participating in the ISTAPS study.

Four doctors (2 males, 2 females) and one female nurse, all with more than 10 years of professional experience, agreed to record their office MI sessions and to recruit smokers. Before beginning the ISTAPS study and during that study period, the health professionals in the intervention group attended 20 hours of workshop training on smoking cessation interventions. The workshops used techniques such as roleplaying and included a four-hour training session in the practical aspects of the MI protocol. In addition, participants attended eight hours of reinforcement sessions [39].

Patients were recruited to the ISTAPS study if they identified themselves as smokers in response to a question from the attending health professional when they came to the PHC office for any reason. Patients who provided informed consent were invited to make another appointment at the office, when the PHC professional collected personal and smoking habit data (selection interview). At the end of the selection interview, smokers identified as being at the precontemplation or contemplation stage of change were interviewed for about 10 minutes, using the brief MI format of incorporating personalized motivating elements into the conversation, based on the Rollnick & Butler model [43]. They were also given a leaflet containing motivational information and told about the help available to them if they changed their minds and decided to quit smoking [39].

The strategy used to select the smokers included in this study was maximum variation sampling [44]. Selection criteria were sex (male–female), age (young-adult-elderly), socioeconomic status [45], low motivation to quit smoking (<5 points according to the Richmond Test score) [46] being in the precontemplation or contemplation stage in the change process (precontemplation-contemplation-preparation-action) [47]. In addition, patients were selected if they agreed to their office visits being recorded for a period of six months. Nine ISTAPS participants met these inclusion criteria. Two of the nine participants came to the office for a second visit during the study period because of a health issue, and at the end of the visit the health professional took the opportunity to conduct a second MI session, for a total of 11 interviews conducted by the five participating health professionals. The characteristics of the nine smokers interviewed are presented in Table 1.

**Table 1:** **Characteristics of smokers with low motivation**

Code	Age	Sex	Socioeconomic status	Cigarettes/ day	Time to first cigarette of day	Smoking-related diseases	Psycho-tropic drugs	Motivation to quit	Stage in process of change	MI
01	22	F	III Non-manual qualified	20	<5 Min	No	No	03 Low	Contemplation	1
02	25	F	I Bachelor’s degree	15	>60 Min	No	No	02 Low	Precontemplation	1
03	25	F	III Non-manual qualified	12	31 to 60 Min	No	No	03 Low	Precontemplation	1
04	28	M	II Self-employed	8	6 to 30 Min	No	No	03 Low	Precontemplation	2
05	43	F	III Non-manual qualified	12	6 to 30 Min	No	Yes	03 Low	Precontemplation	2
06	44	F	IV Partially qualified	8	>60 Min	No	No	00 None	Precontemplation	1
07	50	F	II Self-employed	25	6 to 30 Min	No	No	03 Low	Precontemplation	1
08	62	M	III Non-manual qualified	5	31 to 60 Min	No	No	02 Low	Precontemplation	1
09	49	F	III Non-manual qualified	15	31 to 60 Min	No	Yes	03 Low	Contemplation	1

MI sessions were conducted during 2006 in the Barcelona metropolitan area. The recordings (4 hours, 11 minutes) were transcribed following established CA recommendations [48, 49]. Data were managed using Atlas/ti.5.1. The analysis process (Table 2) was two-fold:

**Table 2:** **Analysis process**

Activity	Researcher
1. Qualitative content analysis of the Motivational Interviewing protocol/guide to identify the actions	R1
2. Construction of a guide for analysis	R1, R2, R3, R4 and R5
3. Reading of transcribed interviews to identify themes	R1
4. Coding of the data by themes, supported by Atlas-ti software	R1
5. Independent reading and identification of analysis categories	R1 and R2
6. Discussion, definition and agreement on codes and categories	R1, R2, R3, R4 and R5
7. Recoding using agreed-upon codes and categories with support from Atlas-ti software	R1
8. Discussion and revision of codes and categories	
Discussion of the data interpretation	R1 and R2 R1, R2, R3, R4 and R5

(R: researcher).

- Qualitative Content Analysis (QCA) of the intervention’s action protocol ISTAPS study identified the actions that needed to be taken during a visit (Table 2, point 1). The ISTAPS research team reached consensus on the meaning of these actions and generated an analytical guide for the video recordings. This analysis allowed us to develop a framework for coding the transcriptions (Table 2, point 2).
- Conversation Analysis (CA) consisted of analysing in detail the semantic content, interactional effects, and consequences observed in the selected sequences corresponding to the study objectives [24]. In our study, nonverbal behaviours were not analysed. The procedure involved coding by topics –structure of the office visit and the actions taken (Table 2, point 4)– and identification of categories according to the different ways of conducting the MI (Table 2, point 5). The process of coding and analysis was recursive; the codes and categories were selected for coherence with the study objectives. (Table 2, points 6 and 8). A total of 106 conversation sequences were analysed.

To ensure the rigour and quality of the study, we based it on the following criteria [50–54]:

- CA was selected as the research methodology because it is focussed on what “happens” in the interaction and “how”. (criterion: epistemological and methodological appropriateness).
- The context for each interview was described (place, interference or interruptions, climate), taking these elements into account in the analysis. Participant selection was done intentionally, with the goal of achieving maximum variation in the sample to ensure generalizability. Recordings were repeatedly played while the transcribed text was read and reread, in order to catch nuances of the interaction. The analysis was carried out independently. All members of the research team have extensive experience in smoking cessation interventions and reviewed the findings carefully, providing feedback to ensure that the results were consistent with the study objectives.
- The study findings were illustrated with specific, relevant sequences that support the interpretations of study results. (criterion: validity).
- The research team reflected on the entire process of the study, including their assumptions and the possible impact on study results, and discussed the role of the professional, various smoking cessation intervention models, and the difficulties patients face when they try to quit smoking (criterion: reflection).

The Ethics and Clinical Research Committee of the Jordi Gol Institute of Research in Primary Care approved the project. Participants were informed that the focus of the research was the doctor-patient relationship in discussions about smoking, and provided signed informed consent that included permission for audiovisual recording of the interviews. Confidentiality was ensured by the coding of participant data. The transcripts were anonymous but linked to the participant code, providing a context (age, sex, etc.) for comments selected from the verbatim transcripts.

## Results

The results were classified into two categories, organization of the motivational interviewing sessions and Professional MI session Practices and Actions, and subcategories with descriptive examples.

### Organization of the MI sessions

This category explains what takes place in the interaction between the health professional and the smoker with low motivation to stop, how it happens and why. In the 11 interviews analysed, the conversation has three phases: assessment, reflection, and summary.

Assessment: The professional begins the MI session according to the intervention protocol of the ISTAPS study, summarizing the data collected in the selection interview (tobacco use, motivation and stage of change). The summary establishes a rapport based on shared understanding and verifies the patient’s readiness to change and to initiate the conversation. This phase of the protocol lasts about three minutes.

Reflection: This phase is central to the MI encounter and requires the most time. In all of the conversations analysed, this phase was initiated by the health professional with a question, such as “why do you think that it is not important for you to quit smoking?” or “can you tell me why you don’t think you could quit smoking?” In each case, the smoker had a chance to express his or her concerns and the health professional noted the individual’s current consumption, statement of positive and negative aspects, and level of intent to change. The conversation was built around these data provided by the smoker. The health professional asked questions, offered information, and affirmed the doubts expressed by the smoker (e.g., “I see, there is a lot of smoking going on in your surroundings and that makes it more difficult for you to quit”). All patients showed some ambivalence about their smoking, with no difference between those in the precontemplative and contemplative stage of change. This phase lasts about five minutes.

Summary: The professional ends the conversation by reviewing the topics covered (e.g., “you told me that you don’t feel prepared to make a quit attempt”) and offering help in the event that the patient wants to quit smoking. The end of the MI encounter is always initiated by the health professional and the smoker responds. This phase lasts about two minutes, for a total average interview length of 10 minutes. During the different phases of the MI session, the interaction is constructed and, despite a standard organizational structure, different interactional dilemmas arise that are resolved in different ways in each interaction and therefore have an effect on the MI encounter.

### Professional MI session practices and actions

This analysis reveals how professionals construct different types of MI encounters and identifies communication that actively centre the conversation either on the patient’s or the professional’s perspective. Interpersonal skills, use of language and application of the ISTAPS study protocol represent distinct social realities reflected in two very different types of practice: patient-centred vs. problem-centred (i.e., the professional uses resources oriented toward resolving the problem, a familiar clinical interaction for the person with a health concern). When patient-centred practice is the dominant aspect of the MI, it is possible to think and talk about tobacco use. If the professional focuses instead on smoking as a health problem, the interview will not achieve its motivational objective. Of the 11 interviews analysed, the predominant practice was problem-centred in seven interviews and patient-centred in only four.

Actions that illustrate these two types of practices are analysed below. The selected actions are patterns of interaction that show the key action that either facilitates or hinders the patient’s reflection on his or her readiness to change in the patient-professional interaction.

### Actions that facilitate reflection on readiness to change

#### Use of reiterations, declarations and open-ended questions

All of the MI sessions included actions that led to a reflection about smoking, although at different levels of intensity, and the health professionals used them in all phases of the protocol. These actions allow the patient to take an active role and build the narrative about his or her use of tobacco.

In this example (the third interview in the analysis), a woman states that smoking is harmful to health but she does not have the confidence to quit. The professional uses different strategies to examine the patient’s problematic situation in depth and helps her reflect on her tobacco use.

Extract 1

1. HP3: … and you say that you aren’t very confident about quitting and…
2. P3: No, more than anything it’s because my partner is a smoker.
3. HP3: Yes…
4. P3: When I’m with him, I smoke more than when I’m not with him, and it’s more difficult for me…

Later:

1. HP3: Anyway, you went 15 days without smoking, that’s really good!
2. P3: Yes
3. HP3: And these 15 days, what happened?
4. P3: Well, it was a special situation. He left, and I was a little depressed and didn’t leave the house.

At the end of the conversation:

1. HP3: You’ve said that you don’t feel ready to try quitting yet.
2. P3: Not right now, mostly because of my partner.

The example starts with a sequence in which the professional reviews an important point that the patient has stated, demonstrating modal reiteration or reflection. The reiteration (1) allows the patient to expand upon the situation and further develop her thoughts (2 and 4). Later, the professional uses a reflection followed by an affirmation, to express support and approval of a smoking cessation attempt (5) and also elicits a consent response (6).

This open-ended question obligates the patient to answer and reflect on what has happened (7 and 8). The conversation ends with another modal reiteration by the professional (9), which allows the patient to consciously explain the reasons for not quitting (10).

These actions (reiteration, declaration, and open-ended questions) allow for a patient-professional interaction that is oriented towards letting the patient reflect on her position. Together with the health professional, the patient constructs a “relationship framework” focused on her own daily life and individual concerns.

### Actions that do not facilitate reflection on readiness to change

Even when professionals take actions to help a patient reflect on smoking, they also use other interaction styles to resolve interactional dilemmas that do not follow MI principles.

### Focusing the conversation on the risks of smoking

In all of the MI sessions, the smokers explained their reasons for smoking and their intention to continue. In four interviews –all of them in the problem-centred professional practice group– the medical professional responded with expert medical advice warning about the risks of smoking. This stance produced an interaction that ignored or blocked the Voice of the Lifeworld. If this style of interaction persists, the intervention is not motivational.

In the second example, a woman is not motivated to quit smoking because she smokes only a few cigarettes a day.

Extract 2

1. HP6: … it can cause cancer,
2. P6: … you can get cancer just because! I had an athletic uncle, he didn’t smoke, he didn’t drink, he had a set sleep schedule, and he had the bad luck of getting cancer and died in two years.

Later:

1. HP6: But you know that you have a higher risk of having health problems!
2. P6: Yes, and if I get in the car and get on the highway, I have a higher risk, ha-ha. And also if I stay at home.

At the end of the conversation:

1. HP6: Just know that even if you don’t smoke a lot, you are harming yourself.

In the first declaration, the professional focuses on the health risks of smoking (1), provoking resistance in the woman, who has another way of thinking about the professional’s declaration of risk (2). This creates a discord in the interaction The professional and the patient address various meanings of the act of smoking; however, the professional does not leave space for any personal reflection on how a meaning applies to the individual patient’s situation, which would help to ensure that, by communicating with the patient, a shared understanding of that meaning has been achieved (3–5). The professional’s interpretations are perceived by the patient as an exercise of power over her discourse, provoking resistance.

This excerpt illustrates the lack of agreement between professional and patient on the existence of a problem. The professional implicitly interprets the patient’s attitude as resulting from a lack of information and attempts to provide details of the possible problems that could arise. However, the patient rejects the arguments because this meaning has not been negotiated and is not shared. When the patient has low motivation to stop smoking, the health professional has an interactional dilemma that he or she resolves by talking about the risks associated with smoking. Focusing the conversation on risk provokes an interaction in which each participant is speaking about different concepts of risk. Misunderstandings arise that make it difficult to build a framework for a shared relationship and easy to move away from MI principles. In the MI approach, information about health risks should be used when the patient asks about them or shows interest in obtaining this information [17].

### Clinging to the protocol

Clinging to the protocol is one way of resolving the interactional dilemma the professional faces when the smoker states his or her intention to continue smoking. This action occurred in seven interviews, all of them dominated by problem-centred professional practice. The professional turned to the intervention protocol to resolve the dilemma and responded by asking a question from the protocol that allows him or her to take control of the conversation.

In this example, the professional diligently follows the MI protocol with a male smoker, but when confronted with a dynamic and complex situation he resolves it rather mechanically by using the ISTAPS study protocol form as a guide.

Extract 3

1. HP4: Yes, zero, that would be no importance, and “10” is the maximum importance. Between zero and 10, where would you place your level of importance to quit smoking right now?
2. P4: Right now? (a pause of 6 seconds). Well, right now it’s zero.
3. HP4: And in the same vein, if you decide to quit smoking right now, what level of confidence (a pause of 15 seconds) would you have right now in quitting? Zero is no confidence and “10” is completely confident.
4. P4: A six.
5. HP4: And how about your readiness to quit right now? Zero is not ready at all (2 seconds’ pause) to quit right now.
6. P4: A six.

When this professional encounters a patient who expresses, from the beginning, little interest in quitting (1–2), there is an opportunity to explore why he has so little motivation. Instead of making an effort to gain an understanding of the patient’s perspective, the interviewer carefully follows the structured sequence of data points, inquiring about confidence and readiness (3–6) to do something the patient has expressed no interest in doing. In this interaction, the professional’s opportunity to explore the patient’s possible ambivalence or other potentially important factors is lost. The patient’s low motivation to stop smoking causes a new interactional dilemma for the health professional. Clinging to the protocol is a strategy that blocks the “Voice of the Lifeworld”; the conversation goes forward but does not necessarily follow the principles of the MI.

### Prematurely emphasizing change

Premature emphasis [55] consists in stressing a behaviour change when the patient has not expressed his or her clear intention to change and occurred in five of the interviews dominated by problem-centred professional practice. This action occurred after the patient expressed some reason to stop smoking or described a previous cessation attempt. The professional grasped onto the declaration and proposed a behaviour change, without taking into account other information the smoker had provided during the conversation.

The selected example shows a man who is motivated to change, but not immediately because of anxiety that is sufficiently severe to require treatment with tranquilizers. In the conversation, the patient explains that the major obstacle to smoking cessation is tobacco dependence in the morning.

Extract 4

1. P9: I don’t know, when I tried quitting that week, I had a really hard time.
2. HP5: Maybe it’s because you tried quitting without any help, don’t you think? I assure you that with a little bit of help it would be better, because you have a high level of dependence -- your nicotine score is quite high -- so that’s why the first two or four weeks would be hard for you without any help, and there are methods.
3. P9: The worst time was the mornings. I can’t.
4. HP5: Yes, yes, mornings are the worst for those with the highest dependence because that’s when they need it the most, which is why I think it would be worth it if you tried again with some treatment.

Later:

1. P9: But I had a really hard time.
2. HP5: You had a really hard time, but it was a week.
3. P9: Yes, and the mornings were the worst.
4. HP5: Yes
5. P9: The afternoons weren’t that bad, I was calm by then.
6. HP5: Sure, if we had some way to alleviate this a bit, how would that be?
7. P9: I think I could quit.

At the end of the conversation:

1. HP5: Do you think that works for you?
2. P9: Well, really it’s the morning.
3. HP5: Above all it’s the morning. Okay, what I’ll do is: I’ll give you a pamphlet that I have here, read it and if, and if you feel like thinking about it, the important part is that you think about which day you would be able to pick a quit date. Would you be able to pick a date or do you want to think about it?
4. P9: I’d rather think about it.
5. HP5: Think about it. Okay. The important part is to think of a date, take a look at a calendar, and make an appointment with me a few weeks before your quit date, okay?
6. P9: I’d rather think about it.
7. HP5: Think about it. Okay. What is important is to think of a date, take a look at a calendar, eh?, a few days or a couple of weeks in advance you make an appointment with me, okay?
8. P9: Mm-hmm (sound of affirmation).

The professional emphasizes what is needed for change too soon and does not ask why the patient’s most recent attempt to quit failed (1–4). The professional believes that the problem is that the patient tried to quit without help. Premature emphasis and lack of exploration into the problem prevents advancement in the reflection process. Rather than explore (beyond what the health professional knows to be true) why it is so hard for the patient to quit smoking in the morning (7–11), the interviewer resumes the conversation without negotiating the next step. MI session has shifted towards the professional’s goal, without using strategies such as reflective listening and further development of the issue. Furthermore, the professional does not address the patient’s use of tranquilizers and the effect this might have on breaking the smoking habit (12–19). The CA of this sequence shows how the health professional confronts a new interactional dilemma. In order to advance the interview, a decision is made to ignore the patient’s “Lifeworld” experience.

## Discussion

### Main findings

Our study has three main findings. The first is that, despite a similar structure in all of the MI encounters analysed (assessment-reflection-summary), we identified different professional practices used to motivate a patient to quit smoking. One of these resembles the Miller-Rollnick model [11], in which interaction is centred on ambivalence toward change. Our results also concur with other reports indicating that these strategies favour a patient-focused interaction [32, 37, 56, 57]. The second practice is a directive interaction, without negotiation and agreement on the existence of a problem, led by the professional and producing hostile or brief answers from the patient and silences from both participants.

The second main finding is that CA shows the complexity of constructing an interaction with a patient whose motivation to stop smoking is low. In order to avoid a confrontation, in which the conversation would become a professional challenge, the health professional must adapt to the patient’s declarations of reasons not to quit smoking. Studies of CA acknowledge that the patient-health professional interaction is collaborative by nature, and also recognize the difficulty in constructing a personalized and negotiated process [30, 36, 58].

Although all participating health professionals attended four-hour training sessions, differences were seen in implementation of the MI sessions. These could be related to the appearance of new interactional dilemmas due to low patient motivation and an accompanying lack of interest in the MI session. Some actions taken to resolve these dilemmas -such as confronting non-negotiated problems, clinging to the protocol, or prematurely emphasizing willingness to change- shift the MI session towards the professional. This often triggers a defensive patient response and/or results in lost opportunities to help the patient reflect on the smoking habit itself. Francis et al. [56] affirmed that professionals tend to enhance confrontational behaviours when the patient has a high resistance to change, making the interaction difficult. Coleman et al. [35] reported that when a patient presented smoking-related health problems, the doctor took a more directive approach. The conversation was focused on the health problem without considering the patient’s point of view, producing confrontational interactions that made it difficult to advance the conversation.

As demonstrated by the different results reported in CA studies, agreement on the existence of a problem is necessary at the beginning of the interaction to avoid hostile responses. Equally important is the way in which health professionals follow up on concerns expressed by the patient; this follow-up facilitates supportive, patient-centred relationships [30, 35, 59, 60]. According to Parry [61], these CA findings have been achieved in the academic sphere and must now be incorporated into training in patient communication offered to all health professionals.

The third main finding is that CA reveals various types of interaction that show how the “Voice of the Lifeworld” and “Voice of Medicine” are used during the MI conversation [32, 62, 63]. The interactional dilemmas with which health professionals are confronted are often resolved using biomedical logic, or the “fix the disease” model. The professional and the patient speak exclusively in “the Voice of Medicine”; “the Voice of the Lifeworld” is ignored or blocked out by the professional. Although health professionals take an interest in having a motivational conversation, the “fix the disease” model persists. This might be explained by adherence to the institutional roles of patient and health professional during the office visit and their interactions are constructed around a health problem to be resolved (diagnosis, treatment, follow-up). It would be interesting to conduct further study of the impact on a normal office visit that could be achieved if both the health professional and patient spoke in “the Voice of the Lifeworld”.

### Strengths and limitations of the study

Several strengths of this study should be highlighted. First, the methodology was an innovative approach, contributing to the CA literature an analysis of MI sessions with low-motivation individuals. These results complement and help to explain, in part, the results of the ISTAPS clinical trial, which found no significant differences between the intervention and control groups in patients who were in the precontemplation stage of change [40]. Secondly, the study demonstrates that CA is a useful approach to analysing the fidelity to MI principles [11, 64] observed in the conversations studied. This is an important strength because of the limited evidence available on this topic [14].

Four potential study limitations should be considered. The present analysis included 11 interviews that illustrate different MI practices. A larger sample could help to identify a wider range of practices and develop a better understanding of how the interaction between the health professional and patient is organized during a MI session about smoking in the PHC setting. Nonetheless, non-motivating patterns of interaction predominated at different points in the conversation during seven of the MI sessions involving three health professionals.

Another possible study limitation is that the MI conversation was conducted in the clinical setting with smokers who had a low motivation to quit, during a visit focused on a health concern and not specifically or solely on smoking. This may have affected the dynamics of implementing a MI session about smoking cessation. On the other hand, the study data were collected in the typical context of the MI.

A third limitation is that classic CA insists on “taking into account” all of the details of the interaction. Although our transcripts substantially followed Atkinson & Heritage, they are somewhat less exhaustive and did not permit context-rich analysis, including intonation, body language, and other nonverbal elements.

Finally, the voluntary participation of the health professionals could have generated bias because these participants were actively interested in smoking interventions, in using the MI session, and in improving this clinical technique. Other professionals who are less interested in this technique would likely follow other MI practices.

### Recommendations for clinical practice

The study findings suggest the following processes that may be advisable to implement in clinical practice:

Before beginning a MI conversation:

- Be aware that the least favourable situation for the MI about smoking cessation involves smokers with low motivation to change that behaviour; this increases the complexity of the intervention.

MI At the beginning of the interview:

- Summarize the information you have about the individual’s tobacco use. Adjust that information as the patient indicates and begin the conversation with an open-ended question, such as “do you feel OK about how much you smoke?” It is not recommended to ask a question to which the obvious correct answer is “stop smoking”.

As the conversation develops:

- Provide continuing feedback to the patient.
- Ask open-ended questions and incorporate the “Lifeworld” voice into the conversation.
- Align the information provided as a health professional with relevant personal concerns expressed by the patient.

## Conclusions

This study underlines the importance of the methods and procedures used by professionals in their patient interactions during a MI encounter. Our analysis suggests that when a health professional encounters individuals with low motivation for change, this increases the complexity of the intervention and several interactional dilemmas may occur that make it difficult to follow basic MI principles. Different forms of expression (reiterations, declarations, open-ended questions) during the MI session could be enough to build a patient-centred relationship. Clinging to the protocol (whether a suggested interview protocol or the process involved in treating the health problem), focusing on risk, or not following up on the patient’s expressed concerns makes it more difficult for health professionals and patients to construct the essential “shared understanding” that allows them to take advantage of the opportunity to reflect on tobacco use during the office visit. Although health professionals take an active interest in having a collaborative relationship, they resolve the dilemmas of interaction from a biomedical perspective.

The study shows that CA is a valid approach to analysing fidelity to MI principles. Therefore, it is important to incorporate the findings of CA studies into professional preparation and practice.
